# Transmission of Onychomycosis and Dermatophytosis between Household Members: A Scoping Review

**DOI:** 10.3390/jof8010060

**Published:** 2022-01-06

**Authors:** Aria Jazdarehee, Leilynaz Malekafzali, Jason Lee, Richard Lewis, Ilya Mukovozov

**Affiliations:** 1 Department of Medicine, University of British Columbia, Vancouver, BC V6T 1Z4, Canada; ariajaz@student.ubc.ca (A.J.); leilynaz@student.ubc.ca (L.M.); dgl@student.ubc.ca (J.L.); 2 Kamloops Dermatology, Kamloops, BC V2C 2H3, Canada; dickiderm@shaw.ca; 3 Department of Dermatology and Skin Science, University of British Columbia, Vancouver, BC V5Z 4E8, Canada

**Keywords:** onychomycosis, tinea, tinea unguium, household, family, environment, transmission

## Abstract

Onychomycosis is a common fungal infection of the nail, caused by dermatophytes, non-dermatophytes, and yeasts. Predisposing factors include older age, trauma, diabetes, immunosuppression, and previous history of nail psoriasis or tinea pedis. Though many biological risk factors have been well characterized, the role of the environment has been less clear. Studies have found evidence of transmission in 44% to 47% of households with at least one affected individual, but the underlying mechanisms and risk factors for transmission of onychomycosis between household members are incompletely understood. A scoping literature review was performed to characterize and summarize environmental risk factors involved in the transmission of onychomycosis within households. A total of 90 papers met the inclusion criteria, and extracted data was analyzed in an iterative manner. Shared household surfaces may harbor dermatophytes and provide sources for infection. Shared household equipment, including footwear, bedding, and nail tools, may transmit dermatophytes. The persistence of dermatophytes on household cleaning supplies, linen, and pets may serve as lasting sources of infection. Based on these findings, we provide recommendations that aim to interrupt household transmission of onychomycosis. Further investigation of the specific mechanisms behind household spread is needed to break the cycle of transmission, reducing the physical and social impacts of onychomycosis.

## 1. Introduction

Onychomycosis is a broad term that encompasses all fungal infections of the nail, including those caused by dermatophytes, non-dermatophytes, and yeasts [1,2,3]. There are multiple subtypes, including distal/lateral subungual, superficial white, and proximal subungual onychomycosis, among others [4]. Clinical features may include nail discoloration, subungual hyperkeratosis, and onycholysis [1]. Left untreated, complications include local pain, paresthesias, spread of infection, as well as the functional and social impairments of nail dystrophy [1,5]. The incidence of onychomycosis is estimated at about 6.5% amongst Canadians, and onychomycosis is thought to be responsible for approximately 50% of nail disorders globally [6].

Dermatophytes account for 90% of onychomycosis cases, and tinea unguium is a term which refers specifically to a dermatophyte infection of the nail [4,7]. Common causative agents include *Trichophyton rubrum*, *T. interdigitale*, and *Epidermophyton floccosum* [1,4]. In children, infection with *T. tonsurans* is frequently seen [4]. Though all dermatophytes can cause onychomycosis, infection due to *Microsporum* spp. is rare [4].

The pathophysiology of dermatophyte infection involves adhesion to the stratum corneum, followed by invasion into the underlying sublayers [8]. Adhesion is facilitated by fibrils on fungal spore cell walls which anchor to host keratinocyte membranes, as well as carbohydrate-specific adhesins which recognize mannose and galactose on host cells [8,9,10]. Following adhesion, spores germinate, forming hyphae that grow in multiple directions, including deeper into the stratum corneum which results in the destruction of subungual structures [9]. The invasion process is facilitated by fungal proteases that hydrolyze extracellular matrix proteins including keratin and collagen, whose breakdown in turn provides nutrients for invading dermatophytes [8,9,11].

Predisposing factors for onychomycosis include older age, trauma, diabetes, immunosuppression, and previous history of nail psoriasis or tinea pedis (athlete’s foot) [1]. Exposure to humid environments, occlusive footwear, and occupations which involve frequent travel, handwashing, or communal bathing facilities increase the risk of developing onychomycosis [7,12]. Studies have found that certain human leukocyte antigen class II genes may influence susceptibility to developing onychomycosis [13,14,15]. Though many biological risk factors have been well characterized, the role of the environment has been less clear. Of particular interest are the factors that influence transmission of onychomycosis between household members. Previous studies have suggested the risk of onychomycosis transmission in households with one affected member to be between 44% and 47% [16,17]. Indeed, using molecular techniques, it was shown that individuals within the same household were infected by the same dermatophyte strain, suggesting likely household transmission [16,18]. Proposed mechanisms of transmission between household members include sharing of slippers or even walking on carpets or bathroom floors previously walked on by an affected individual harboring infectious fungal elements [19]. Indeed, studies have found that dermatophytes are able to survive in washed textiles such as socks and contaminated nail polish containers [20,21]. Despite this, studies directly implicating environmental risk factors to transmission of onychomycosis are lacking, and more evidence is required to understand social determinants associated with the development of onychomycosis.

In light of this knowledge gap, we conducted a scoping literature review to characterize and summarize the environmental risk factors involved in the transmission of onychomycosis between individuals within households. A better understanding of the factors contributing to the spread of onychomycosis within households will allow for the development of recommendations which aim to limit household spread. Given the high incidence of onychomycosis and the functional and societal impacts of infection, our findings may play a significant role in reducing the incidence of infection through primary prevention.

## 2. Materials and Methods

Given the paucity of studies directly investigating the spread of onychomycosis among household members, we conducted a scoping literature review for broad assessment of evidence relevant to the topic. A scoping review allows for the systematic mapping of research done in a broad context [22]. Like systematic reviews, scoping reviews require structured, comprehensive searches to produce reproducible results [22]. The protocol for this scoping review was drafted in accordance with the Preferred Reporting Items for Systematic Reviews and Meta-analysis Extension for Scoping Reviews [23].

### 2.1. Search Strategy, Study Eligibility Criteria, and Study Selection

A systematic search of the literature was conducted by combining search terms for onychomycosis and disease transmission using the MEDLINE database (United States National Library of Medicine) through the OVID interface. The search strategy is outlined in Table 1. Studies were deemed eligible for inclusion if they were published in peer-review journals, written in English, and published between 1950 and 2021. Human studies were prioritized, and both primary studies and reviews were included. In vitro and animal studies were excluded.

The final search results were transferred to COVIDENCE (Melbourne, Australia) (www.covidence.org; last accessed on 1 November 2021) for title, abstract, and full-text screening [24]. Three reviewers (A.J., L.M., J.L.) independently evaluated titles, abstracts, and full texts to identify relevant studies. Disagreements were resolved by consensus and through discussion with the senior reviewer (I.M.).

### 2.2. Data Extraction and Synthesis

Three reviewers (A.J., L.M., J.L.) independently extracted data from eligible studies using a standardized extraction form that included title, authors, year of publication, study population, and key findings. All reviewers evaluated the extracted data and worked together to synthesize the available evidence in an iterative manner. As part of the scoping review methodology, supplemental searches were done to support the findings of our initial literature search. These additional searches were guided by reviewing reference lists of included studies to ensure that all primary studies relevant to our search were captured.

## 3. Results

The search yielded 425 results, 90 of which were deemed relevant for review. Of these, 49 were primary studies, 32 were review papers, and 9 were mixed-method studies. The date of the studies ranged from 1954 to 2020 and they included data on both adult and pediatric populations.

### 3.1. Transfer through Shared Surfaces

Dermatophytes have been found to persist on a variety of surfaces, particularly wet surfaces on which individuals walk barefoot. Multiple species, including *T. rubrum* and *T. mentagrophytes* have been isolated from walkways, changing rooms, and foot washing stations in swimming pool facilities, despite regular disinfection with chlorine [25]. Dermatophytes were found to be in greatest concentrations along walkways where people converged, including entrances and exits [26]. Aside from swimming pools, studies have found *T. rubrum*, *T. mentagrophytes*, and *T. tonsurans* on the floors of mosques, wrestling mats, and nursing homes [27,28,29]. In one nursing home study, *T. tonsurans* was isolated from 22.8% of samples collected from various bedroom and bathroom surfaces [30]. Clinically relevant species of dermatophytes have even been isolated from garden soils and beach sand which have been in contact with human feet [31].

Multiple studies have shown that these surfaces may be not only harboring dermatophytes, but also driving transmission of onychomycosis. In an investigation of an outbreak in a long-term care facility in which patients were mostly bedridden with minimal direct interpersonal contact, transmission was attributed to a shared bathtub, in which 9 strains of *T. interdigitale* were found [29]. In another study, the same dermatophyte species were found within house dust on the floors of affected individuals in 48 of 117 (41%) cases [32]. Raboobee et al. found a higher prevalence of tinea pedis and unguium among individuals who had visited a mosque compared to a control group, and isolated various yeasts and *Trichophyton* spp. from carpets walked on by affected individuals [33].

The use of slippers or other footwear not shared with other household members may limit dermatophyte spread from shared surfaces. In one study conducted in an Italian military school where individuals wore sandals while showering, only 0.2% of those tested were found to have onychomycosis [34].

In summary, shared surfaces may harbor dermatophytes and provide sources of infection. Applying these findings to the household environment, dermatophytes may likely be found on wet surfaces, such as patios, balconies, washrooms, showers, and bathtubs. There may also be a high density of dermatophytes in areas of convergence, such as entrances and narrow hallways. Care should be taken to properly disinfect and avoid walking barefoot on these surfaces.

### 3.2. Transfer through Shared Equipment

Aside from shared surfaces, shared equipment may also play a role in the transmission of onychomycosis among family members. Multiple studies have implicated use of shared hair equipment, including combs, hairbrushes, and headrests with the transmission of *T. rubrum* and *T. mentagrophytes*, causing tinea capitis [30,35,36]. One study conducted in the context of a barber shop found dermatophytes and non-dermatophytes, primarily *Aspergillus* spp., on 24.4% of combs, hairbrushes, shaving brushes, and chair headrests tested [35].

Other studies investigating dermatophytosis in general have suggested that sharing soaps, towels, bedding, and general articles of clothing may be implicated in dermatophyte transmission, and individuals are at greatest risk when there is any skin trauma or breaks in skin barrier [37,38]. Though this data relates to dermatophyte spread in general, it is clinically relevant for development of onychomycosis because studies have shown that 30% of cutaneous dermatophyte infections have nail involvement [39]. Development of these infections, particularly tinea pedis, has been established as risk factors for developing onychomycosis [39,40].

Relating more specifically to the feet and toenails, studies have cited sharing of sandals, slippers, and footwear as risk factors for transmission of onychomycosis. Indeed, *E. floccosum*, *T. rubrum*, and *T. mentagrophytes* have been found on the surfaces of slippers, sandals, and socks in multiple studies [41,42,43,44]. Methods such as ultraviolet radiation and ozone have been shown to reduce fungal burden on these types of footwear [44].

Sharing of nail polish may also promote transmission of onychomycosis. One study found that *T. rubrum* is able to survive in nail polish for 60 days at 25 °C [21]. There have been no studies examining fungal burden on other nail equipment such as nail clippers, files, or scissors, but these may also be implicated with spread. Nail trauma, including micro traumas following manicures or pedicures, may increase the risk of infection with these shared tools [45].

### 3.3. Persistence of Fungi within the Household Environment

In addition to the mechanisms of fungal spread discussed above, the persistence of organisms within the household environment may promote reinfection or provide sources of infection for other household members. Indeed, dermatophytes have been found to persist in the household environment for up to 18 months [46].

Multiple studies have demonstrated the ability of fungi to persist on household cleaning supplies. Ekowati et al. found 15 clinically relevant fungi species on 21 of 24 (88%) samples of cleaning supplies, including mops, scrubbers and wipes [26]. In many cases, the same species on the cleaning supplies were also isolated from floors, and the authors argue that improperly disinfected cleaning tools may promote fungal spread [26]. Other studies have found *M. gypseum* and *T. mentagrophytes*, common agents of onychomycosis, in up to 48.4% of household vacuum cleaners tested [47,48], and it has been argued that vacuums without proper filters may result in further dissemination of fungi and spores [49].

Textiles, particularly clothing and bedding, may also harbor fungal pathogens, and studies have previously implicated textiles with human infection [38,50]. The warm environment and presence of desquamated keratinocytes provides for ideal growing conditions for fungi [51]. Multiple studies have shown persistence of dermatophytes on clothing, particularly socks, despite regular laundering [20,44]. Indeed, yeast and fungi have been shown to withstand washing temperatures below 40 °C and 60 °C, respectively [52,53]. This has implications for cold-water laundering, and studies have also shown that inadequately cleaned washing machines spread fungi to previously sterile textiles [54,55].

Pets may also play a role by harboring fungi, and pet ownership has been cited as a risk factor for developing onychomycosis [56]. In one study, contact with cats or dogs was reported in 39.5% of those with dermatophytosis [57]. *M. canis* has most commonly been reported in cats and dogs, and human dermatophyte infections have been directly linked to these animals [58,59]. Small rodents such as guinea pigs have been known to harbor dermatophytes [60]. In one study of 101 guinea pigs with dermatophytosis, 98 (97%) were positive for *T. mentagrophytes* and 24% of households had family members with clinical signs of dermatophytosis, with greater involvement in children than adults [61]. There have also been multiple reported outbreaks of *T. benhamiae* and *T. rubrum* in humans attributed to small rodents, including guinea pigs and rabbits, through genotyping techniques [60,62,63]. In many cases, pets were brought into the household just weeks prior to development of clinical symptoms in adults, which reinforces the need to properly examine new pets entering the household to prevent introduction of fungal pathogens [61]. In summary, dermatophytes are able to persist within the household environment, namely on cleaning supplies, textiles, and pets.

## 4. Discussion

It is well established that individuals with onychomycosis are at risk of spreading infection to other household members. The proportion of households demonstrating spread of onychomycosis following infection of an initial member has been found to be between 44% and 47% [16,17]. Molecular techniques have provided further support for household spread by identifying the same strains of dermatophytes in affected members of a household [16,18]. Though theories behind household transmission have been suggested, there have been no studies formally investigating the mechanisms behind transmission. Our findings suggest that shared surfaces, shared equipment, and persistence within the household may present opportunities for reinfection and spread of onychomycosis among household members.

The findings of our review suggest that indirect transmission, rather than direct contact, between household members plays a key role in the spread of onychomycosis within households. We did not find any data implicating direct human–human transmission within the household. Interestingly, the prevalence of infection appears to be higher between spouses and children compared to those marrying into the family, highlighting the genetic predisposition underlying infection [15]. Aside from genetic predisposition, household members with diabetes, psoriasis, poor peripheral circulation, or immunosuppression, or smoking habits may be at greater risk of developing infection [7].

**Figure 1:**
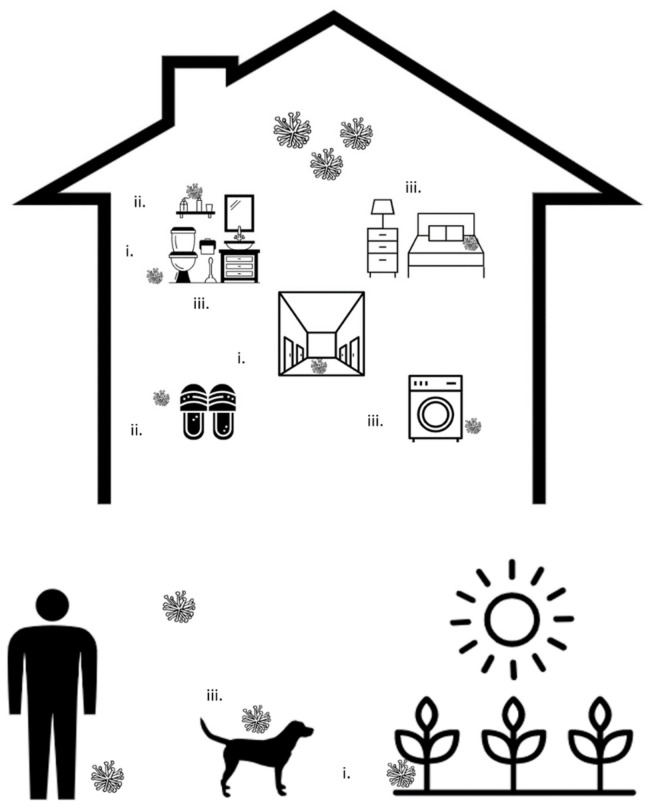
Summary of potential pathways involved in the household transmission of onychomycosis: (**i**) Shared surfaces within the household may harbor fungi and provide sources for transmission. Possible surfaces include patios, balconies, washrooms, showers, bathtubs, and areas of convergence such as entrances and hallways; (**ii**) Shared household equipment, including footwear, bedding, and nail tools, may facilitate transmission of onychomycosis; (**iii**) Fungi may also persist within the household environment, on cleaning tools, linen, and pets, serving as ongoing sources of infection.

Our study findings of the possible mechanisms for household transmission of onychomycosis are summarized in Figure 1. Based on these findings, a list of recommendations that aim to interrupt transmission of onychomycosis in the household setting is proposed and summarized in Table 2. In addition to these recommendations, measures should be taken at the individual level to prevent infection, including maintaining foot hygiene and wearing non-occlusive shoes. Many of these recommendations are summarized in table 6 of Gupta et al. [7].

Breaking the cycle of household dermatophyte transmission may not only prevent onychomycosis, but also limit asymptomatic carriage which may predispose one to onychomycosis. One study found that household members are able to asymptomatically carry and spread dermatophytes for up to two months [36]. Children may play a key role in asymptomatic transmission, and it has been argued that children are more likely to harbor dermatophytes due to close contact with floors and soils [47,64]. One study found dermatophytosis to be asymptomatic in 16% of children [64], and another found dermatophyte carriage in 86% of family members with one affected child with tinea pedis [65]. Recent advances in the diagnosis of onychomycosis may facilitate the detection of cases in asymptomatic individuals in order to better characterize patterns of transmission among household members [66].

Given the paucity of studies directly exploring the mechanisms behind household transmission of onychomycosis, this study is limited to a broad overview of factors which may contribute to this spread. A limitation to our findings is that they are based on studies conducted on transmission of other dermatophytes resulting in human infection at other body sites, such as tinea capitis. This evidence is clinically relevant, however, given the similar mechanisms of dermatophyte infections across body sites. Other limitations reflect the scoping review design, and include the lack of study quality appraisal and possible selection bias of the papers included in the analysis.

## 5. Conclusions

Our study highlights several factors which may facilitate the persistence and spread of onychomycosis among household members. Further investigation of the specific mechanisms behind household spread is needed to break the cycle of infection and reinfection, which would reduce the physical and social impacts of onychomycosis.

## Figures and Tables

**Table 1:** MEDLINE search strategy.

Search	Keywords
**1**	Onychomycos*
2	tinea unguium
3	Dermatophyte*
4	disease transmission, infectious
5	transmission
6	household
7	1 or 2 or 3
8	4 or 5 or 6
9	7 and 8

**Table 2:** Recommendations to limit the household spread of onychomycosis.

**Minimizing Spread from Shared Surfaces**
Properly disinfect floors and bathtubs, particularly in high traffic areas (e.g., entrances, hallways)
Wear socks or slippers around the house
Regularly wash feet and wipe them dry
**Minimizing spread from shared equipment**
Minimize sharing of slippers or socks, and disinfect slippers regularly
Minimize sharing of nail polish and nail tools (clippers, scissors, files)
Disinfect nail tools before and following use
**Minimizing persistence of dermatophytes in the household environment**
Use single-use cleaning supplies or regularly disinfect cleaning tools to prevent the spread of fungi
Launder textiles at temperatures > 60 °C
Regularly disinfect laundry machines
Maintain hygiene of pets
